# The Empire Dental Sterilizer

**Published:** 1897-05

**Authors:** 


### Editorial, Etc.

THE EMPIRE DENTAL STERILIZER.—Wilmont Castle & Co., Rochester, N. Y.—The necessity for sterilizing dental instruments to prevent transmitting disease germs from one patient to another is so well recognized at the present time that a good sterilizer becomes a necessasy appliance in a dental office.

The Empire Dental Sterilizer, which appears to fully meet such an indication, consists of an oval metal drum 7¾ inches long, 5¾ inches wide and 6½ inches high, and divided perpendicularly into two compartments. In the upper part of one end is a movable pan designated to hold borophenol, an antiseptic fluid, one ounce of which in eight ounces of distilled water will make a 4 per cent. solution of boracic acid. This solution is well adapted for use in the sterilizer. The other end of the apparatus is adapted to contain anti-septicated sand which is thoroughly washed in a solution of boracic acid, then dried and finally trituated with boro phenol. This sand is heated from 200 to 250 degrees F. For operating the sterilizer, pour one ounce of boro phenol and eight ounces of distilled or boiled water into the movable pan. Partly fill the other compartment with the antisepticated sand and place a small burner or lamp under the sand end. Dip the instruments to be sterilized in the solution for a few minutes (5 or 10), then draw them back and forth through the heated sand, and they will become bright, clean and perfectly sterilized. Small instruments such as burs, root drills, clamps, etc., are to be placed in the strainer provided, when they are immersed in the solution for 5 or 10 minutes, when the hot sand is scooped up with the strainer a few times and let run through on the instruments.

